# Emergence of a methicillin-susceptible *Staphylococcus aureus* ST672 clone associated with invasive paediatric infections in Mexico

**DOI:** 10.3389/fcimb.2026.1796701

**Published:** 2026-05-15

**Authors:** David Juárez-Hinojos, Jocelin Merida-Vieyra, Laura Belmont-Monroy, Isaac Alberto Vigil-García, Nancy Evelyn Aguilar-Gómez, Agustín De Colsa-Ranero, Ma. Guadalupe Aguilera-Arreola, Alejandra Aquino-Andrade

**Affiliations:** 1 Laboratory of Molecular Microbiology, Instituto Nacional de Pediatría, Mexico City, Mexico; 2 Laboratory of Medical Bacteriology, Instituto Politécnico Nacional, Mexico City, Mexico; 3 Department of Infectious Diseases, Instituto Nacional de Pediatría, Mexico City, Mexico

**Keywords:** MSSA, paediatrics, PVL, SCV, ST672, WGS

## Abstract

**Introduction:**

*Staphylococcus aureus* sequence type (ST) 672 is a pathogenic clone first identified in Asia, associated with invasive infections in healthy individuals and outbreaks in hospitals and the community. In Mexico, this clone was reported in 2022 as a cause of osteoarticular infections in children; however, its virulence and resistance profiles remain unknown. Therefore, the aim of this study was to phenotypically and genomically characterize *S. aureus* ST672 isolates obtained from invasive paediatric infections at a tertiary-care hospital in Mexico.

**Methods:**

Eight *S. aureus* ST672 isolates from patients at the National Institute of Paediatrics (INP) were included. Antimicrobial susceptibility testing and the induction of small colony variants (SCV) phenotype were performed. Whole-genome sequencing was carried out using Illumina technology. Bioinformatics tools were used to identify resistance and virulence genes, as well as mutations in their regulatory regions and mobile genetic elements. Twenty-five genomes from public databases were included for phylogenetic comparison. The clinical course of patients was described.

**Results:**

All *S. aureus* ST672 isolates were methicillin-susceptible and showed a reversible SCV phenotype; the *bla*Z gene was detected in six isolates. Adhesins *atl, efb, ebp, fnb*A/B*, sdr*C/D/E, *ica*A/B/C/R were identified in all isolates, as well as *agr* operon type I. The *pvl* gene was detected in six isolates. Additionally, five carried a mobilizable plasmid, pN315-like. All strains harboured an intact ϕSa6-like prophage. The *luk*SF-PV operon was found in the ϕSa2/ϕSa2-like prophage. Mexican strains formed a single clade and, unlike other genomes, carried the *luk*SF-PV. Seven infections were community-acquired and showed no evidence of a local outbreak.

**Conclusions:**

The methicillin-susceptible *S. aureus* (MSSA) ST672 clone represents a community lineage with genomic features associated with invasion and emergent persistence in Mexico and shows the capacity to adapt to the hospital environment. These findings support the need for genomic surveillance of MSSA clones.

## Introduction

*Staphylococcus aureus* is a Gram-positive bacterium that colonizes about 30% of humans and causes diseases such as skin and soft tissue infections (SSTI), bloodstream infections (BSI), endocarditis, pneumonia, osteoarticular infections (OAI), and infections associated with medical devices (Urish and Cassat, 2020; Masters et al., 2022; Touaitia et al., 2025). This bacterium has adhesins and immune evasion factors such as protein A (*spa*) and the immune evasion cluster (IEC) ΦSa3, as well as cytotoxins including α-haemolysin and Panton-Valentine leukocidin (PVL). It can also form biofilms. Additionally, *S. aureus* is characterized by the adoption of persistent phenotypes that allow it to survive in tissues and on orthopaedic implants (Masters et al., 2022; Tuon et al., 2023). Methicillin-susceptible *S. aureus* (MSSA) strains exhibit significant phenotypic diversity (Urish and Cassat, 2020; Wong Fok Lung et al., 2022).

The main invasive diseases in children include BSI, OAI, pneumonia, and severe SSTI, which are associated with longer hospital stays, invasive procedures (such as abscess drainage, surgical debridement, and amputation), and orthopaedic sequelae (including pseudoarthrosis and chronic pain) (Urish and Cassat, 2020; Masters et al., 2022). Although research has mainly focused on methicillin-resistant *S. aureus* (MRSA), surveillance studies have shown that MSSA is the leading cause of invasive paediatric infections across multiple settings (DeLeo et al., 2010; Kao and Fritz, 2025).

In 2019, *S. aureus* was associated with 1.1 million deaths across 135 countries, demonstrating that this bacterium causes the highest number of infectious deaths worldwide. According to these data, 48.1% of cases were due to lower respiratory tract infections, 27% to BSI, 15.3% to peritoneal and intra-abdominal infections, and 3% to and subcutaneous infections (GBD 2019 Antimicrobial Resistance Collaborators, 2022).

In the US, the incidence of invasive MSSA is 1.8 times that of MRSA (31.3 vs. 17.5 per 100,000 inhabitants), and MSSA accounts for ~60% of cases and deaths from invasive disease, affecting individuals without comorbidities or previous hospital exposures, including children (Jackson et al., 2020). In Australia, a multicentre study involving 57 institutions documented 3,422 episodes of *S. aureus* BSI, of which 83.9% were MSSA. In addition, 77% of cases were community-acquired (Coombs et al., 2024).

In 2017, a Latin American multicentre study including nine hospitals characterized 1,185 *S. aureus* isolates from BSI, of which 55% were MSSA. These strains remained susceptible to most antibiotics tested, except for erythromycin (E) and tetracycline. A predominance of MSSA in BSI has also been reported across several countries in the region, including Argentina, Chile, Colombia, and Ecuador (55–78%) (Arias et al., 2017).

Consistent with this regional trend, data from Mexico also indicate an increasing predominance of MSSA. The drug resistance surveillance research network (INVIFAR) reported a rise in MSSA from 50% to 75% between 2009 and 2018 (Garza-González et al., 2020). This pattern has likewise been observed in other national collections, including BSI isolates (62%) (Vazquez-Rosas et al., 2021) and OAI in the paediatric population (92%) (Aguilar-Gómez et al., 2022).

Pulsed-field gel electrophoresis (PFGE), multilocus sequence typing (MLST), *spa* typing, the staphylococcal chromosomal cassette *mec* (SCC*mec*), and the presence of PVL are useful for lineage assignment. Whole-genome sequencing (WGS) is the method of choice for outbreak resolution, phylogenetic reconstruction, and characterization of the resistome and virulome. Gene-by-gene schemes (cgMLST/wgMLST) and phylogenomic approaches based on single-nucleotide polymorphisms (SNPs) can reveal microevolution in hospital and community settings, detect transmission events, and identify accessory genome determinants linked to clinical outcomes (Echaniz-Avilés et al., 2025).

*S. aureus* sequence type 672 (ST672), belonging to clonal complex 361 (CC361), has been increasingly reported since its first description in 2012 in South Asia. It has been identified across South and West Asia, as well as in parts of Oceania, and has been detected in both MSSA and MRSA, frequently associated with *spa* type t3841 (Khedkar et al., 2012; Balakuntla et al., 2014; Sarkhoo et al., 2021). These reports highlight the genomic plasticity of ST672, particularly its capacity to acquire SCC*mec* elements and other mobile genetic elements, reflecting its evolutionary adaptability.

Genomic analyses of ST672 isolates have revealed a diverse accessory genome comprising prophages, plasmids, and virulence-associated determinants. In particular, the presence or absence of IEC-associated genes, PVL, and resistance determinants such as *mec*A and *bla*Z suggests a dynamic mobilome that may contribute to variation in virulence, colonization, and antimicrobial susceptibility. Additionally, phenotypic variability, including differences in biofilm-forming capacity, has been documented and may contribute to persistence and colonization in both infected and asymptomatic carriers (Locke et al., 2024).

Recent epidemiological studies have further expanded the known distribution of ST672. In a prospective cohort study in Sri Lanka, ST672 was identified in both clinical infections and colonizing isolates (25%, 22/88), predominantly as MSSA, with *spa* type t3841 highly prevalent. Notably, most isolates were PVL-negative but exhibited variable biofilm-forming capacity, including strong biofilm production in colonizing strains, suggesting a potential role in persistence and transmission dynamics (Locke et al., 2024). Similarly, large-scale genomic surveillance in Saudi Arabia has identified ST672 among the dominant sequence types in *S. aureus* populations (7.67%, 47/612), although it remains less prevalent than globally disseminated clones such as ST5, ST8, ST30, and ST22 (Alhejaili et al., 2025).

In Mexico, ST672 was first reported in 2022, when it was identified as the most common sequence type (18.5%) among paediatric OAI (Aguilar-Gómez et al., 2022). It has since been linked to severe skin and soft tissue infections in paediatric patients, further underscoring its clinical relevance in invasive disease (Key et al., 2023). However, despite these reports, the genomic and phenotypic characteristics of ST672 in invasive paediatric infections remain poorly understood, particularly in Latin America.

Taken together, available evidence suggests that ST672 is a genetically adaptable and geographically expanding lineage of *S. aureus* with variable virulence and resistance profiles. Nevertheless, comprehensive studies that integrate clinical data, phenotypic characterization, and whole-genome analysis remain limited. In this context, the present study aimed to characterize ST672 isolates associated with invasive paediatric infections by integrating genomic, phenotypic, and clinical data to better understand their epidemiological and biological relevance.

## Materials and methods

### Location of the study

A retrospective, descriptive, single-centre study was conducted at the National Institute of Paediatrics (INP), a tertiary-care hospital located in southern Mexico City, with 251 beds and approximately 6,592 hospital admissions per year (Instituto Nacional de Pediatría, 2024).

### Bacterial isolates

Through institutional monitoring systems in the Pediatric Intensive Care Unit (PICU) (INP 2023/013) and the *OsteoCode* program (INP 2019/007) (Aguilar-Gómez et al., 2022) at INP, conducted from January 2019 to July 2024, a total of 26 MSSA isolates were identified from paediatric patients with invasive infections. Isolates were recovered as part of routine microbiological surveillance from OAI (n=20) and paediatric intensive care unit samples (n=6).

### Susceptibility profile

Susceptibility to cefoxitin (FOX), clindamycin (CM), gentamicin (GE), E, trimethoprim/sulfamethoxazole (SXT), ciprofloxacin (CIP), and linezolid (LZD) was determined using the disc diffusion method, following the Clinical and Laboratory Standard Institute M100 document (CLSI, 2024).

### Induction and characterization of SCV

To determine the presence of the SCV phenotype, the protocol described by Loss et al. (2019) was followed, with some modifications. The strains were inoculated into brain heart infusion (BHI) medium (Becton Dickinson, Cuautitlán Izcalli, Mexico) and incubated at 37 °C for 24 h. From this culture, a suspension equivalent to 0.5 McFarland (1.5x10^8^ CFU/mL) was prepared in 0.85% saline solution. This suspension was diluted to 1:10,000 in isotonic saline, and 100 µL were inoculated in triplicate by the spread plate method onto 5% sheep blood agar (ASC) (Becton Dickinson, Le Pont-de-Claix, France) and tryptic soy agar (TSA) plates (Becton Dickinson, Le Pont-de-Claix, France).

The plates were then incubated at 37 °C for 24 h. The images of the plates were captured and analysed using ImageJ (v1.5) to measure colony size. Colonies with a diameter less than one-fifth of the median, showing loss of pigmentation and haemolysis, were classified as SCV. These colonies were subcultured in ASC and TSA. Stable SCV were defined as those that did not grow on TSA and maintained the phenotypic characteristics of SCV on ASC. In contrast, reversible SCV were those that recovered haemolysis, pigmentation, and colony size (Loss et al., 2019).

### Genomic analysis

Genomic DNA for WGS was extracted using the Monarch^®^ Spin gDNA Extraction Kit (New England Biolabs, Ipswich, USA) following the manufacturer’s instructions. DNA quantification was performed by fluorometry with a Qubit 4 dsDNA HS Assay Kit (Invitrogen, Eugene, USA). The samples were sequenced on an Illumina NovaSeq 6000 system with paired readings of 150 bp.

The quality of the readings was evaluated using FastQC v0.11.9 (Babraham Bioinformatics-FastQC A Quality Control tool for High Throughput Sequence Data). *De novo* assembly was performed using SPAdes v4.0.0 (Prjibelski et al., 2020), and assembly quality was assessed using QUAST v5.0.2 (Gurevich et al., 2013).

Resistance genes were identified using ResFinder v4.6.0 (Camacho et al., 2009; Bortolaia et al., 2020) and the Comprehensive Antibiotic Resistance Database (CARD) (Alcock et al., 2023). Virulence factors were identified with the Virulence Factor Database (VFDB), considering only genes with an identity greater than 90% (Chen et al., 2005). The search and characterization of plasmid replicons were performed using MobSUITE (Robertson and Nash, 2018), and Pairwise Sequence Alignment to obtain identity percentage was done with EMBOSS Needle (Madeira et al., 2024). Genome annotation was performed using SnapGene v8.1 and Prokka v1.14.6 (Seemann, 2014). The spaTyper v1.0 server was used for *spa* typing (Bartels et al., 2014), and the *agr* operon type was determined with AgrVATE v1.0.2 (Raghuram et al., 2022).

The assembled genomes were analysed using the Phage Search Tool Enhanced Release (PHASTER), and prophage regions were identified based on homology to phage databases and the presence of characteristic structural genes, including integrase, terminase, capsid, tail proteins, holin, and endolysin (Zhou et al., 2011; Arndt et al., 2016).

To identify SNPs linked to virulence regulation and the SCV phenotype, the genomes were compared with the reference *S. aureus* NCTC8325 genome (RefSeq NC_007795.1). The reference genome was indexed using bowtie2-build v2.5, and read mapping was performed with Bowtie2 v2.5.4 (Li et al., 2009; Langmead et al., 2019). Alignments were limited to regions defined in a browser extensible data (BED) file using SAMtools view. Variants were identified with BCFtools v1.22 (Danecek et al., 2021) using the *mpileup/c* strategy. Only positions with a minimum reading depth (DP) ≥30× and a quality score (QUAL) ≥30 were considered. Functional annotation was performed on the Galaxy platform (Afgan et al., 2018) using SnpEff v5.2 and SnpSift v5.2 (Cingolani et al., 2012); annotations were exported to tables and filtered by variant type and predicted impact.

### Phylogenetic comparison

To position the INP strains within the global landscape of the ST672 lineage, a comparative phylogenetic analysis was conducted using the eight isolates from this study and 25 genomes from public databases (John et al., 2023; Che Hamzah et al., 2024). Assemblies in FASTA format included information about the country of origin. Additionally, metadata such as to clinical source, year of isolation, and infection type were recorded when available.

A core genome alignment was generated using Parsnp v2.1.5 (Kille et al., 2024) and processed with HarvestTools v1.3 (Treangen et al., 2014). The resulting alignment was used to create a maximum-likelihood phylogenetic tree in IQ-TREE v2.4.0 (Minh et al., 2020) using the GTR+G substitution model, with 1,000 bootstrap replicates to evaluate branch support.

The final phylogenetic tree was visualized and annotated using iTOL v5 (Letunic and Bork, 2021), incorporating information on geographic origin, source of isolation, resistance profile, virulence factors, and *spa* type obtained using the methodology described above.

### Clinical data

Electronic clinical records were reviewed retrospectively to collect demographic data. Patients were categorized by age as neonates (less than 29 days), infants (under 1 year), children (over 1 year and under 12 years), or adolescents (over 12 years and under 18 years). Comorbidities were classified according to a standardized scheme of complex chronic conditions (congenital malformations, chromosomal abnormalities, endocrine, metabolic, nutritional, mental and behavioural disorders, non-neoplastic haematological disorders, haematological malignancies, and solid tumours; neurological disorders, genitourinary disorders, immunodeficiencies, dermatological disorders, and musculoskeletal disorders), as well as hospitalizations within the 12 months prior to the episode (Lindley et al., 2020).

For each infectious event, the infection type and source (hematogenous, contiguous focus, catheter-related, or pulmonary) were recorded. The classification of infection acquisition was as follows: community-acquired infection (CAI), defined as onset before 48 hours of hospitalization without healthcare exposure, or hospital-acquired infection (HAI), defined as occurring on or after calendar day 3 of hospitalization (Centers for Disease Control and Prevention, 2025).

The laboratory parameters included were those obtained upon hospital admission: erythrocyte sedimentation rate (ESR), white blood cell (WBC) count, and C-reactive protein (CRP) level. For ESR, reference values were 0–10 mm/h for patients ≤12 years; for those >12 years, values were 0–15 mm/h in males and 0–20 mm/h in females. ESR was considered elevated when it exceeded the upper limit for age and sex. Total leukocytes count (10³/µL) varied by age (6–10 years: 4.5–13.5; 10–14 years: 5.0–11.0; ≥14 years: 4.5–11.0), and leukocytosis was defined as values above the upper limit of the corresponding interval. CRP levels ≤0.8 mg/dL were considered normal, whereas values >0.9 mg/dL were considered elevated (Ahsan et al., 2024).

Medical interventions included the use of invasive devices (e.g., central venous catheter or prosthesis), mechanical ventilation, surgical procedures performed during hospitalization, length of hospital stay, and transfer to the PICU.

Empirical treatment was defined as the antibiotic administered at the start of symptoms, before susceptibility results were available, while definitive treatment was guided by microbiological findings. The following information was recorded: whether antimicrobial management involved monotherapy or combination therapy, changes in treatment, total duration of therapy, and whether oral treatment was indicated at discharge (Woods et al., 2021).

Clinical outcomes were reviewed and classified as clinical cure, relapse/recurrence, or death. Clinical cure was defined as the resolution of signs and symptoms of infection, no need for additional antibiotics at the end of antimicrobial therapy, improvement at the first outpatient follow-up visit, and negative control cultures when indicated (Woods et al., 2021). Relapse/recurrence was defined as a new clinical episode with isolation of *S. aureus* at the same site within 90 days after completing treatment (Chong et al., 2013; Russell et al., 2024).

### Declaration of ethics

This study was conducted using isolates obtained from two protocols approved by the Research (17 CI 09 003 109), Biosafety (17 СВ 09 003 143), and Ethics (CONBIOETICA-09-CEI-025-20161215) committees of the Instituto Nacional de Pediatría (IRB: 00008064; IRB: 00008065) with registration numbers 2025/041, INP 2019/007, and INP 2023/013. For the INP 2019/007 project, informed consent was obtained from the parents or legal guardians, and informed assent was obtained from children older than 8 years. For the INP 2023/013 project, the ethics committee waived the requirement for informed consent because the isolates were obtained as part of standard care, and the conduct of this study did not interfere with patient clinical management. Both protocols were conducted in accordance with the principles of the Declaration of Helsinki, and all patient data were de-identified.

## Results

### Bacterial isolates and sequence typing

ST30 and ST152 were each identified in three cases, and ST5 and CC1 were each identified in two cases. Additional sequence types were represented by single isolates, including CC1221, ST7222, ST188, ST3994, ST15, ST25, ST9, and ST1159. The sequence type ST672 was detected in 30% (n=8) of the 26 MSSA isolates.

### Phenotypic characteristics of *S. aureus* ST672

**Figure 1:**
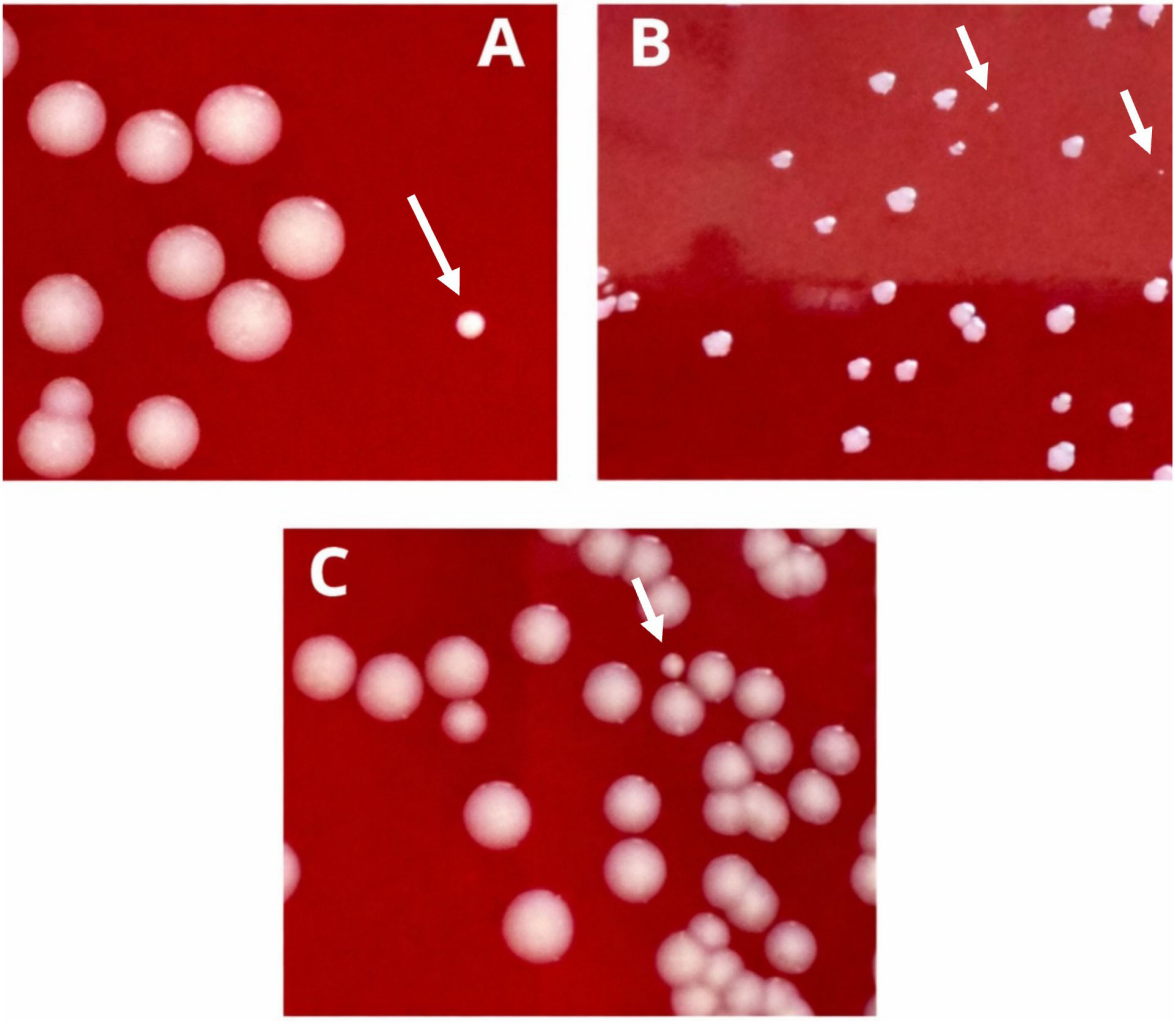
Induction of SCV phenotype. Panels **(A)** (O43), **(B)** (UTIP55), and **(C)** (UTIP77) show representative photographs of colonies displaying the small colony variant (SCV) phenotype. Arrows indicate SCV colonies.

Six isolates were susceptible to all the antibiotics tested, whereas strains O55 and UTIP77 were resistant to GE. Reversible SCV phenotype was detected in all eight isolates (**Figure 1**).

### Genomic analysis

Among the eight *S. aureus* genomes analysed, the assemblies ranged in contig number from 13 to 25. The N50 values varied between 274,955 bp and 696,889 bp, whereas L50 values ranged from 2 to 4. The G+C content was highly conserved across all strains, spanning a narrow range of 32.68% to 32.76% (**Supplementary Table 1**).

**Figure 2:**
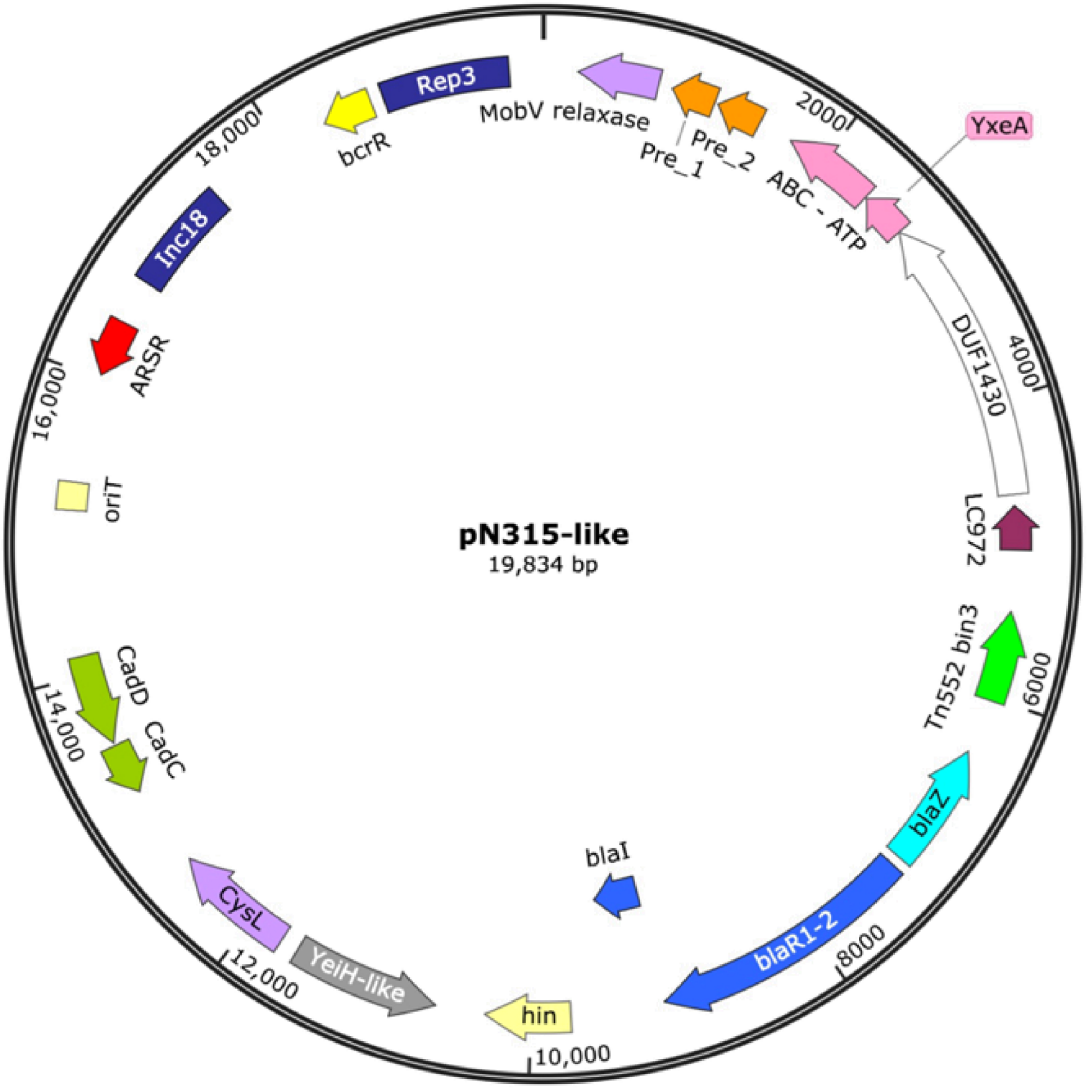
Circular map of the pN315-like plasmid (19,835 bp) identified in *S. aureus* ST672 isolates. MobV (relaxase) and Pre_1 and Pre_2 (plasmid recombination enzymes). ABC-ATP (putative ATP-binding protein with ABC transporter function), DUF1430 (domain of unknown function conserved in bacteriocins), LC972 (lactococcin protein 972), Tn552 bin3 (putative transposon, with DNA invertase potential), *bla*Z (beta-lactamase), *bla*R1-2 (beta-lactam signal transducer), *bla*I (beta-lactamase repressor), hin (DNA invertase), YeiH-like (putative intermembrane protein related to transport), *cys*L (transcriptional regulator for cysteine biosynthesis), *cad*C and *cad*D (regulatory proteins for cadmium resistance), *ori*T (transfer origin), ARSR (arsenic resistance operon), Inc18 (replication region), *bcr*R (transcriptional activator), and *rep*3 (replication initiator protein).

In five strains (19, O43, O55, O59, and Sa531), a 19,835 bp plasmid was identified, assembled into a single contig, and had a G+C content of 28.3%. This plasmid carried the Rep3 replicon belonging to the Inc18 incompatibility group, cadmium resistance genes, a relaxase of the MOBV family, and a MOBQ transfer origin that was classified as mobilizable (**Figure 2**).

**Figure 3:**
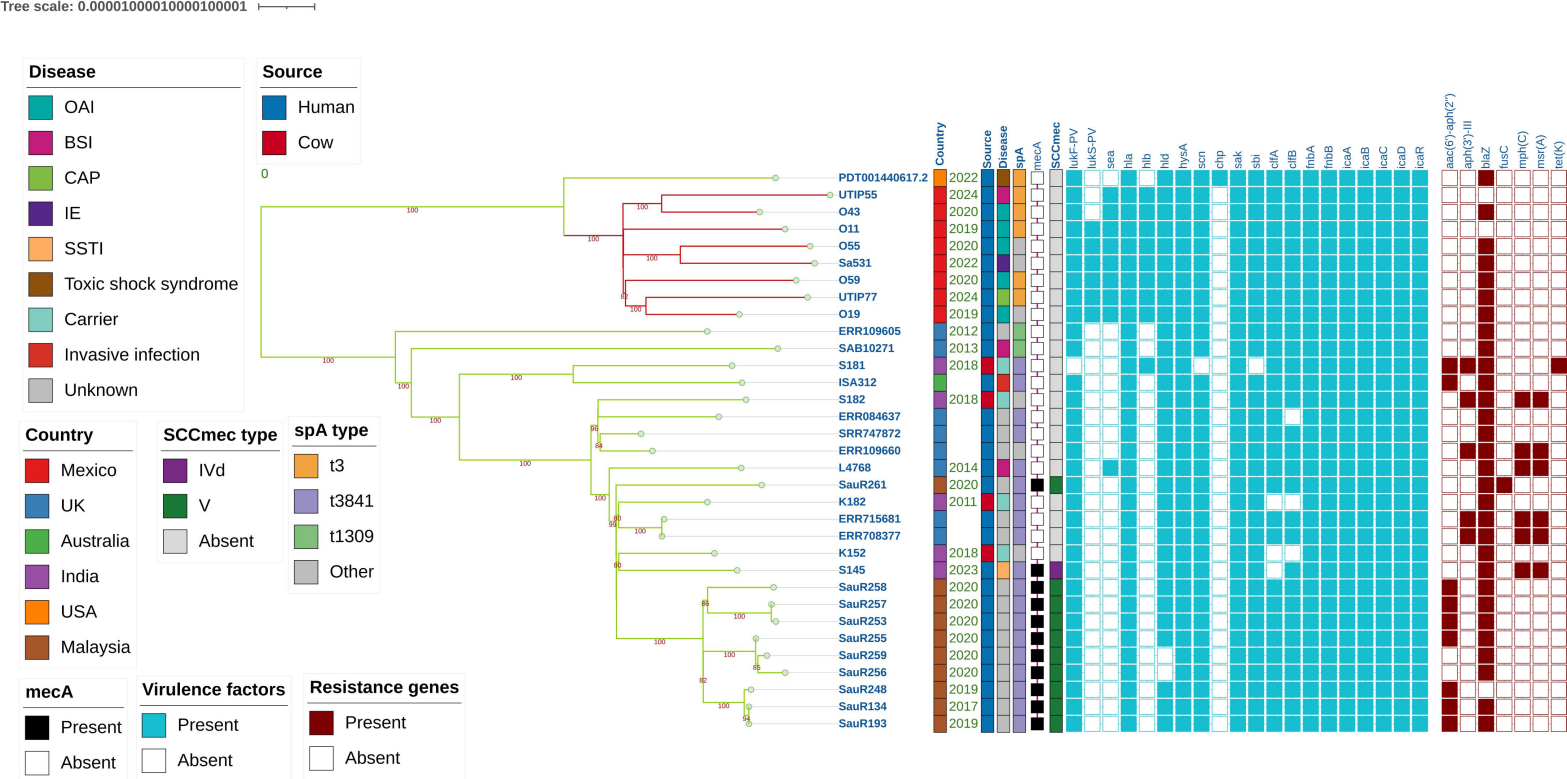
Maximum likelihood phylogenetic tree based on the central genome of 33 genomes of *S. aureus* ST672, including 8 from the National Institute of Paediatrics (Mexico) and 25 from public databases. The tree was inferred with IQ-TREE (GTR+G model, 1,000 bootstrap replicates); bootstrap values ≥70 is shown. The lateral annotations indicate the country of isolation, source, type of infection, *spa* type, presence of *mec*A and SCC*mec*, and binary profiles of virulence factors and resistance genes.

None of the ST672 genomes carried penicillin-binding protein 2a (*mec*A) or other elements of the SCC*mec* chromosomal cassette. Four *spa* variants were identified: t003, t014, t1227, and t4568. All the isolates carried the accessory gene regulator (*agr*) type 1 operon, with no mutations detected in the open reading frame (ORF). Regarding acquired resistance genes, six strains carried beta-lactamases (*bla*Z); in two of these, the aminoglycoside acetyltransferase/phosphotransferase [*aac(6′)-aph(2′′)]* was also detected, whereas two isolates did not carry any acquired resistance genes (**Figure 3**).

Characterization of the virulome revealed that all the strains carried fibronectin-binding protein A (*fnb*A), fibronectin-binding protein B (*fnb*B), alpha-haemolysin (*hla*), delta-haemolysin (*hld*), and the intercellular adhesion genes (*ica*A, *ica*B, *ica*C, *ica*D, and *ica*R). Genes encoding Panton-Valentine leukocidin (*luk*S-PV*/luk* F-PV) were identified in six isolates (**Figure 3**). None of the isolates carried the collagen adhesin (*cna*), leukocidin E (*luk*E), or toxic shock syndrome toxin (*tsst*-1).

Staphylococcal complement inhibitor (*scn*), staphylokinase (*sak*), and staphylococcal binder of immunoglobulin (*sbi*), were detected in all strains, whereas the chemotaxis inhibitory protein (*chp*) was not detected (**Figure 3**, **Supplementary Table 2**).

Prophages ϕSa2, ϕSa2-like, ϕSa6-like, and ϕSa3 were identified. Prophages belonging to the ϕSa2 group were identified in five strains (O11, O19, O55, O59, and UTIP77), all of which carried the *luk* SF-PV operon. Strains O43, UTIP55, and Sa531 carried degenerated ϕSa2-like phages that lacked the *luk* S/F-PV genes. In all strains, at least one intact ϕSa6-like prophage was present. Phages of the ϕSa3 group were only detected as incomplete or fragmented remnants, lacking integrase or IEC genes (*chp*, *sak*, and *scn*).

In global regulators, mutations were observed in *agr*C (histidine kinase sensor of the *agr* system, variants p.V26G and p.Y235F), *rot* (repressor of toxins, p.S126N), *rsb*W (anti-sigma pathway factor *σ^B*, p.V22A), *sar*A (staphylococcal accessory regulator A, p.E121K), *sar*T (adhesin transcriptional regulator, p.D63E), and *sig*B (sigma factor B, p.Q256K).

In two-component systems, mutations were detected in *arl*S (*h*istidine kinase sensor of the ArlRS system, p.E121D), *gra*R (*r*esponse regulator of the GraRS system, p.Q148E and p.Q148H), *gra*S (histidine sensor kinase GraS, p.F26L and p.L59I), *sae*S (sensor of the SaeRS system, p.N227S, p.E268K, and p.D269N), and *srr*B (*s*ensor of the SrrAB system, p.K100M and p.Q235L).

Mutations were identified in metabolism related genes across all genomes, including *hem*A (aminolevulinic acid synthetase, p.T22A and p.T327A), *hem*Y (protoporphyrinogen oxidase, p.F130L, p.L133F, p.D141N, p. N269S, and p.H325Y), *men*C (o-succinylbenzoate synthase, p.A43G, p.A43T, p.A209V, p.A265V, p.E303D, p.A308T, p.H309Q, and p.H309N), *men*D (2-succinyl-6-hydroxy-2,4-cyclohexadiene-1-carboxylate synthase, p.D148N, p.V338I, p.E345K, p.C349R, and p.L375F), and *thy*A (thymidylate synthase, p.K100N). Additionally, in strain Sa531, p.V22A was observed in *rsb*W; strain UTIP55 carried p.S126N mutation in *rot*; and strains O43 and UTIP55 carried p.Q235L in *srr*B.

### Phylogenetic comparison

The isolates from this collection were clustered into a monophyletic clade (bootstrap = 100), clearly separated from the remaining ST672 genomes included in the analysis. Within this Mexican clade, two main subgroupings were observed: (i) a subclade formed by O43 and UTIP55 (bootstrap = 100) and (ii) a subclade including O19, UTIP77, O59, O11, Sa531, and O55, with variable internal support (**Figure 3**).

Genomes from the United Kingdom, India, Australia, and Malaysia were distributed outside the Mexican clade, forming a separate group, with internal structure in which one subclade predominantly grouped genomes from Malaysia (SauR134–SauR261). Isolates from India and the United Kingdom were interspersed at different positions within this clade, whereas the genomes from Australia (ISA312) and India (S181) formed a closely related pair (bootstrap = 100) within the non-Mexican group (**Figure 3**).

The genomes originated mainly from Asia (14/33) and the Americas (9/33). The most frequent *sp*A type was t3841 (19/33), followed by *sp*A t3 (6/33), which was detected exclusively in isolates from the Americas. The *mec*A gene was identified in isolates from Malaysia and India, associated with SCC*mec* type V (10/11) and IV (1/11). Notably, only the Mexican strains carried the *luk*F–*luk*S genes (6/8) (**Figure 3**).

### Clinical data

A total of 75% of patients were in the paediatric age group, and five were male. One patient had complex congenital heart disease with a history of neurosurgery. The most common admission diagnosis was trauma (n=3), followed by community-acquired pneumonia (n=2). Infections were classified as OAI (n=5), endocarditis, BSI, and pneumonia (n=1 each). Seven infections were classified as CAI. The most common source of infection was haematogenous (n=4). The median length of hospital stay was 28 days (range 17–68). For empirical therapy, cephalothin (CF) was administered as monotherapy (n=4). Three patients received CF as definitive treatment, and three received combination therapy with CM. The median duration of antimicrobial treatment was 28 days (range: 16–68). One patient died (**Supplementary Table 3**).

## Discussion

This study reports the emergence of *S. aureus* ST672, a methicillin-susceptible strain carrying the pvl gene, isolated from invasive infections in Mexican paediatric patients. This clone has previously been reported primarily as MRSA in Asia and has also been identified in food products, livestock, and healthy carriers, underscoring its broad ecological distribution and potential for transmission (Khedkar et al., 2012; Sarkhoo et al., 2021; Boswihi et al., 2024).

All ST672 isolates in this study were MSSA. Notably, in both high- and low-income countries, MSSA infections are more prevalent than MRSA among paediatric patients (Jackson et al., 2020; GBD 2019 Antimicrobial Resistance Collaborators, 2022; Prochaska et al., 2023; Rojas-Larios et al., 2023; Coombs et al., 2024).

In the same institution, a previous longitudinal surveillance study of bloodstream infections from 2006 to 2019 did not identify ST672 among the characterized isolates (Vazquez-Rosas et al., 2021). Although sequence typing was not performed on the entire historical collection, these data suggest that ST672 was not detected among previously analysed bloodstream infection isolates, supporting its recent emergence in our setting.

The detection of reversible SCVs across all isolates suggests a transient adaptive state characterized by metabolic alterations and reduced regulation of virulence-factor expression, thereby affecting intracellular survival and bacterial persistence. This phenotype is associated with changes in metabolic pathways, including hemin, menadione, or thymidylate biosynthesis, as well as decreased activity of the *agr* system, resulting in slow growth, reduced toxin production, and increased tolerance to antibiotics (Proctor et al., 2006; Sendi and Proctor, 2009; Guo et al., 2022). Although stable SCVs were not identified in this study, the reversibility of this phenotype is clinically relevant, as reversible SCVs can emerge under selective pressure or antimicrobial treatment and subsequently revert to a normal growth phenotype (Guo et al., 2022).

From a diagnostic perspective, SCVs represent a challenge for clinical laboratories due to their slow growth, atypical morphology, and reduced or absent haemolysis, which can lead to underdiagnosis or delayed identification. SCVs detection is not routinely performed in most clinical laboratories, either because of operational limitations or a lack of knowledge about this phenotype. Nevertheless, several studies have reported associations between the presence of SCVs and persistent or recurrent clinical manifestations, including osteomyelitis, septic arthritis, and infections associated with medical devices, supporting the need for more comprehensive microbiological analyses in patients with these types of infections (Kahl et al., 2016; Keim et al., 2023).

A mobilizable 19.8-kb plasmid, such as pN315, carrying the Rep3 replicon with Inc18, and a relaxase of the MOBV family, was identified in five genomes (O19, O43, O55, O59, and Sa531). This element carries *bla*Z and cadmium resistance determinants that could facilitate horizontal gene exchange between *Staphylococcus* spp. and other Gram-positive cocci (Mores et al., 2021; Al-Trad et al., 2023). The recurrent detection of these plasmids in phylogenetically diverse strains suggests an acquisition or maintenance event, possibly favoured by metal-rich microenvironments. Previous studies have reported that, in *S. aureus* isolates from wastewater, 62% of plasmids carry both *bla*Z and cadmium resistance genes (*cad*D and *cad*X). The Pairwise Sequence Analysis between all plasmid sequences displayed an identity percentage above 99% in all cases, suggesting that this plasmid is highly conserved in the MSSA strains analysed (**Supplementary Table 4**). These plasmids may act as evolutionary reservoirs, enabling genetic plasticity that facilitates the incorporation of resistance or virulence genes, even in antibiotic-susceptible lineages (Amirsoleimani et al., 2021). This study is limited by the use of short-read Illumina sequencing, which may hinder accurate reconstruction of repetitive and mobile genetic elements, including plasmids. In particular, draft genome assemblies may fragment or incompletely resolve genetic structures, potentially affecting the precise determination of their genomic organization. Therefore, the mobilome features described herein should be interpreted as putative genomic inferences rather than fully resolved structural entities. Long-read sequencing approaches would be required to achieve complete plasmid reconstruction.

Despite this limitation, the consistent identification of these elements across phylogenetically related and unrelated isolates supports their potential biological relevance and suggests stable maintenance within the population under study.

The resistome analysis confirmed the absence of *mec*A and SCC*mec* and detection of *bla*Z (6/8) and *aac(6′)-aph(2′′)* (2/8). The presence of *bla*Z reflects historical beta-lactam antibiotic pressure, likely sustained by its empirical use in community-based paediatric infections (Pennone et al., 2022; Kilani et al., 2024). Despite antimicrobial susceptibility, infections caused by these isolates were characterized by prolonged clinical courses, combination therapy, and sequelae, indicating that virulence and bacterial persistence can serve as clinical determinants, alongside antimicrobial resistance (Prochaska et al., 2023; Free et al., 2025).

In characterizing the virulome, a pattern of factors typically associated with persistent community clones of *S. aureus* was observed. The presence of *atl*, *efb*, *ebp*, *fnb*A/B, and *sdr*C/D/E in all genomes, together with the *ica*ADBCR operon, supports adhesion, biofilm formation, and tissue colonization (Peng et al., 2022). To confirm the role of the *ica* operon in this lineage, microscopy-based assays and gene expression analyses would be required. The absence of *cna* distinguishes ST672 from classical clinical lineages and suggests that adaptation toward a persistent phenotype is not dependent on collagen binding. The presence of PVL in 75% of the isolates is consistent with its association with OAI and pulmonary disease and reinforces the hypothesis that the combination of PVL and *ica*ADBCR potentiates deep-tissue colonization and evasion of innate immunity (Masters et al., 2022). Additionally, the pattern of incomplete immune evasion, with the presence of *scn*, *sak*, and *sbi* but the absence of *chp*, is consistent with the presence of truncated ΦSa3 phages. ΦSa2 phages, mostly with homology to Φ*2958PVL* and Φ*7401PVL*, were present in all *pvl-*positive strains, confirming their role in the dissemination of the *luk*SF-PV operon (Coombs et al., 2020). All the strains harboured a ϕSa6-like structural phage that lacked virulence genes and showed homology to Φ11 and Φ80α. This phage is considered part of the basal mobilome of *S. aureus* and has been proposed to facilitate recombination and contribute to genomic plasticity.

In contrast, the absence of a complete ΦSa3 prophage, together with the detection of *sak* and *scn*, suggests loss of this phage and the possible chromosomal integration of IEC-associated remnants (Saei et al., 2024). Given that IEC components have been linked to immune evasion mechanisms, including complement inhibition and interference with neutrophil chemotaxis, their presence may contribute to host adaptation and persistence.

However, the functional impact and expression of these elements were not experimentally assessed in this study. Therefore, these findings should be interpreted as genomic inferences rather than as direct evidence of immune evasion. Further studies integrating transcriptomic and functional approaches will be necessary to determine the role of IEC in the pathogenic potential of ST672.

Moderate-impact mutations were identified in global regulators (such as *agrC, sar*A*, sig*B*, rsb*W, and *rot*) and in two-component systems (including *graRS, srrAB, sae*RS, and *arl*RS). Similarly, substitutions were detected in metabolic genes (*hemA, hem*Y*, men*D*, men*C, and *thy*A) involved in the respiratory chain and in folate biosynthesis (Wong Fok Lung et al., 2022).

These genetic variations may reflect adaptive processes associated with intracellular persistence, metabolic remodelling, or modulation of virulence expression, as previously described in *S. aureus*. However, their functional impact was not experimentally assessed in this study. Therefore, these findings should be interpreted as descriptive and hypothesis-generating, based on genomic inference rather than on confirmed gene expression or phenotypic assays.

Further studies integrating transcriptomic analyses with cellular infection models will be required to determine the contributions of these regulatory and metabolic pathways to ST672’s pathogenic potential and persistence.

Comparative phylogenetic analysis revealed that the ST672 isolates in this collection constitute a distinct sublineage within the global panorama, as evidenced by the aggrupation of a monophyletic clade with maximum support. This clustering suggests that the Mexican strains do not represent multiple recent emergences from other countries but instead correspond to a closely related local lineage, most likely derived from a single introduction event followed by local diversification.

The existence of two well-defined subclades within the Mexican group suggests ongoing microdiversification. consistent with sustained circulation of this lineage in the region. In contrast, the genomes from Asia, Europe, Oceania, and the US clustered outside the Mexican clade, forming a separate and heterogeneous phylogenetic group. Within this global group, a subclade comprising primarily isolates from Malaysia was observed, indicating a specific regional expansion of ST672 in Southeast Asia. The presence of genomes from India and the United Kingdom within this clade supports the hypothesis that ST672 is a cosmopolitan lineage with multiple dispersal events (Derelle et al., 2025). The geographic distribution of the analysed genomes reflects limitations in the availability of sequences in public databases and highlights important differences in genetic profiles. The *spa* t3841 type, which is predominant worldwide, differs from the *spa* t3 type, which is restricted to isolates from the American continent, suggesting a geographical association of the *spa* gene. A particularly relevant finding is that only isolates from Malaysia and India carried *mec*A, associated with SCC*mec* types V and IV, whereas all Mexican strains were MSSA. This finding indicates that methicillin resistance is not an inherent trait of the ST672 lineage but has been independently acquired in specific geographical contexts (Sarkhoo et al., 2021; Boswihi et al., 2024).

The *luk*S/*luk*F genes were found only in the Mexican isolates, reinforcing the hypothesis that this sublineage has acquired specific virulence factors absent from the international genomes analysed. These findings suggest that, in the regional context, ST672 may have followed an evolutionary path focused on virulence rather than resistance, consistent with its association with invasive infections in the paediatric population (Masters et al., 2022).

Five of the eight isolates were obtained from OAI, all of which required antimicrobial treatments longer than three weeks and combinations of antimicrobials. Although six patients were clinically cured without immediate complications, the frequency of sequelae was high: one relapse was documented, and one hospital case (UTIP77) resulted in a fatal outcome. These observations highlight that antimicrobial susceptibility does not guarantee rapid resolution in MSSA infections with persistent virulence (Free et al., 2025). The predominance of CAI, the involvement of previously healthy children (7 of 8), and the diversity of clinical manifestations observed (osteomyelitis, septic arthritis, endocarditis, BSI, and pneumonia) underscore this clone’s invasive and adaptive capacity.

A case-level integration of clinical, genomic, and phenotypic data revealed distinct patterns within the ST672 clone. The fatal case (UTIP77) involved a patient with CAP who required intensive care, mechanical ventilation, and PICU admission. The case was caused by a PVL-positive isolate that also exhibited resistance to GE and was associated with leukocytosis and elevated inflammatory markers, reflecting a systemic inflammatory response.

In contrast, the recurrent case (O59) occurred in an OAI caused by a PVL-positive isolate and was associated with sequelae, despite the absence of identifiable genomic differences compared with non-recurrent cases. Because all isolates exhibited an SCV phenotype, this supports the role of phenotypic adaptation in persistence and long-term clinical impact.

At the genomic level, the high degree of conservation across isolates, including shared variants in global regulatory systems (e.g., *agr*, *sar*A, *sig*B) and two-component systems, suggests a stable clonal background maintained regardless of clinical diagnosis or outcome. These variants were consistently present across isolates from different infection types and disease severities, supporting the notion of a genetically cohesive lineage.

An invasive BSI caused by a PVL-negative isolate was observed in a nosocomial setting involving invasive devices, indicating that PVL is not essential for invasive disease and highlighting the role of host and clinical factors in this patient.

Most OAI cases occurred in previously healthy paediatric patients with CA infections, often associated with trauma, and were characterized by variable inflammatory responses, including leukocytosis and elevated CRP, along with a prolonged disease course and frequent sequelae.

Overall, these findings suggest that clinical outcomes within this clone are shaped by the interplay among conserved genomic features, variable PVL presence, and a shared SCV phenotype. However, given the limited sample size, further studies with larger cohorts are needed to establish statistically robust associations.

Limitations of this study include the relatively small number of isolates and the single-centre design, which limit the generalizability of the findings. Therefore, the results should be interpreted as an initial characterization of *S. aureus* ST672 associated with invasive paediatric infections in a tertiary-care setting.

Despite these limitations, the consistent phenotypic and genomic features observed across isolates support the biological relevance of the findings and suggest the presence of a locally circulating sub-lineage.

No robust association between specific mutations and clinical presentation or outcome could be established. This limitation is mainly due to the small sample size, the lack of statistically powered subgroup comparisons, and the high genomic similarity among isolates, which limits the ability to detect genotype–phenotype correlations. Additionally, the presence of widespread conserved variants across all isolates further limits the discriminatory power for clinical stratification.

In this context, our findings should be interpreted as evidence of the potential emergence of ST672 in invasive paediatric infections, rather than definitive confirmation, highlighting the need for larger, multicentre studies to further characterize and expand these observations.

To our knowledge, this study represents the first genomic characterization of *S. aureus* ST672 associated with invasive infections in the Mexican paediatric population. This study contributes to understanding the adaptive features of this clone and provides evidence that antimicrobial susceptibility does not preclude virulence or persistence.

In conclusion, ST672-MSSA is proposed as an emerging clone in our environment, a community lineage capable of causing invasive infections and prolonged clinical courses, despite its susceptibility to beta-lactams. The detection of an MSSA-PVL positive clone with these traits at a reference hospital serves as a warning and reinforces the need to maintain active surveillance in this setting.

## Data Availability

The datasets presented in this study can be found in online repositories. The names of the repository/repositories and accession number(s) can be found in the article/**Supplementary Material**.
