# Supplementary material for: Emergence of a methicillin-susceptible Staphylococcus aureus ST672 clone associated with invasive paediatric infections in Mexico
Source: Front Cell Infect Microbiol. 2026 May 15;16:1796701. doi: 10.3389/fcimb.2026.1796701 (PMC13219331; doi:10.3389/fcimb.2026.1796701)
Supplement: Supplementary file 1 [file Table1.docx]

Supplementary table S1. Reads and genome assembly statistics of *S. aureus* ST672 isolates obtained from pediatric invasive infections

| Strain | #Contigs | #Contigs  >1000 bp | Largest contig (bp) | Genome size (bp) | N50 | L50 | G+C (%) | N (%) |
| --- | --- | --- | --- | --- | --- | --- | --- | --- |
| O19 | 23 | 19 | 647,546 | 2,786,217 | 309,753 | 4 | 32.73 | 0 |
| O43 | 15 | 14 | 656,330 | 2,743,876 | 607,402 | 3 | 32.73 | 0 |
| O55 | 24 | 21 | 647,212 | 2,757,901 | 274,955 | 4 | 32.68 | 0 |
| O59 | 13 | 12 | 1,260,417 | 2,745,764 | 696,889 | 2 | 32.72 | 0 |
| Sa531 | 19 | 15 | 702,193 | 2,786,121 | 337,903 | 3 | 32.73 | 0 |
| UTIP55 | 25 | 21 | 382,512 | 2,721,842 | 310,224 | 4 | 32.76 | 0 |
| UTIP77 | 24 | 21 | 648,772 | 2,783,541 | 335,069 | 4 | 32.74 | 0 |
| O11 | 25 | 22 | 648,256 | 2,763,661 | 361,615 | 3 | 32.76 | 0 |

Assembly statistics for the eight *S. aureus* ST672 genomes, including total number of contigs, contigs larger than 1,000 bp, largest contig size (bp), total genome size (bp), N50, L50, GC content (%), and percentage of ambiguous bases (N). bp, base pairs; N50, length of the shortest contig among the longest set of contigs that cover 50% of the genome; L50, number of contigs whose cumulative length accounts for 50% of the genome; GC, guanine–cytosine content; N, percentage of ambiguous bases.
