# Supplementary material for: Emergence of a methicillin-susceptible Staphylococcus aureus ST672 clone associated with invasive paediatric infections in Mexico
Source: Front Cell Infect Microbiol. 2026 May 15;16:1796701. doi: 10.3389/fcimb.2026.1796701 (PMC13219331; doi:10.3389/fcimb.2026.1796701)
Supplement: Supplementary file 2 [file Table2.docx]

**Supplementary Table S3.** Clinical, microbiological, and treatment characteristics of pediatric patients infected with *S. aureus* ST672

| **Variable** | **O11** | **O19** | **O43** | **O55** | **O59** | **Sa531** | **UTIP55** | **UTIP77** |
| --- | --- | --- | --- | --- | --- | --- | --- | --- |
| Age | Pediatric | Pediatric | Pediatric | Pediatric | Pediatric | Adolescence | Pediatric | Adolescence |
| Sex | Male | Male | Female | Male | Female | Male | Female | Male |
| Comorbidities | None | None | None | None | None | Neurological, congenital heart disease | None | None |
| Previous hospitalizations | None | None | None | None | None | Yes | None | None |
| Admission diagnosis | Trauma | Trauma | Dog bite | Trauma | Fracture | Cardiopathy | CAP | CAP |
| Leukocytosis | No | Yes | No | Yes | No | Yes | Yes | Yes |
| ESR (mm/h) | 50 | 53 | 56 | 24 | 40 | 94 | 98 | NP |
| CRP (mg/dL) | 0.4 | 33 | 0 | 86 | 0.3 | 14.6 | 15.9 | 19.5 |
| Type of infection | COM | SA | AOM | COM + SA | CO + SA | IE | BSI | CAP |
| Acquisition | CAI | CAI | CAI | CAI | CAI | CAI | HAI | CAI |
| Source of infection | Contiguous focus | Hematogenous | Contiguous focus | Hematogenous | Hematogenous | Hematogenous | Catheter-associated | Pulmonary |
| Invasive devices | None | None | None | None | None | None | Orotracheal intubation, jugular/femoral CVC, urinary catheter | Mechanical ventilation, CVC |
| Hospital stay (days) | 28 | 17 | 17 | 46 | 39 | 68 | 48 | 23 |
| PICU admission | No | No | No | No | No | No | Yes | Yes |
| Initial therapy | CF | CF | CF | CF | CF + CM | VAN | CRO + VAN + LZD + FEP + MEM | CRO, LZD, VAN |
| Definitive therapy | CF + CM | CF | CF | CF + CM | CF + CM | CF + RA | CF | VAN, LZD, MEM, CLR, SXT |
| Monotherapy / combination | Combined | Combined | Monotherapy | Combined | Combined | Combined | Combined | Combined |
| Total therapy duration (days) | 28 | 16 | 17 | 45 | 38 | 68 | 48 | 23 |
| Decesed | No | No | No | No | No | No | No | Yes |
| Sequelae | No | Yes | Yes | Yes | Yes | Yes | Yes | NA |
| Oral therapy (at discharge) | CF | CFR | CM | CFR | CF | Transferred | No | NA |
| Oral therapy duration (days) | 92 | 12 | 14 | 126 | 62 | Transferred | NA | NA |
| Recurrence | No | No | No | No | Yes | Transferred | No | NA |

Laboratory values correspond to the first determination obtained during hospital admission. Oral therapy refers to antimicrobial treatment prescribed at discharge for outpatient continuation. CF, cephalothin; CFR, cephadroxil; CM, clindamycin; VAN, vancomycin; CRO, ceftriaxone; LZD, linezolid; FEP, cefepime; MEM, meropenem; RA, rifampicin; CLR, clarithromycin; SXT, trimethoprim-sulfamethoxazole; ESR, erythrocyte sedimentation rate; CRP, C-reactive protein; CAI, community-acquired infection; HAI, healthcare-associated infection; COM, chronic osteomyelitis; SA, septic arthritis; AOM, acute osteomyelitis; IE, infective endocarditis; BSI, bloodstream infection; CAP, community-acquired pneumonia; NP, not performed; NA, not applicable; CVC, central venous catheter; PICU, pediatric intensive care unit.
