# Supplementary material for: Emergence of a methicillin-susceptible Staphylococcus aureus ST672 clone associated with invasive paediatric infections in Mexico
Source: Front Cell Infect Microbiol. 2026 May 15;16:1796701. doi: 10.3389/fcimb.2026.1796701 (PMC13219331; doi:10.3389/fcimb.2026.1796701)
Supplement: Supplementary file 4 [file Table4.docx]

**Supplementary Table S4.** Individual comparisons of percentage identity between all pN315-like plasmids using Pairwise Sequence Alignment.

| **%Identity comparisson** | | | | | |
| --- | --- | --- | --- | --- | --- |
|  | **O19** | **O43** | **O55** | **O59** | **Sa531** |
| **O19** | - | 99.10% | 99.90% | 99.20% | 100% |
| **O43** | 99.10% | - | 99.10% | 99.60% | 99.10% |
| **O55** | 99.90% | 99.10% | - | 99.20% | 99.90% |
| **O59** | 99.20% | 99.60% | 99.20% | - | 99.20% |
| **Sa531** | 100% | 99.10% | 99.90% | 99.20% | - |
